# Leveraging Synergy: A Review of the Therapeutic Potential of SN-38 and Immune Checkpoint Blockade in Breast and Prostate Cancer Treatment

**DOI:** 10.3390/jpm15110512

**Published:** 2025-10-30

**Authors:** Tayo A. Adekiya, Simeon K. Adesina

**Affiliations:** Department of Pharmaceutical Sciences, College of Pharmacy, Howard University, Washington, DC 20059, USA

**Keywords:** breast cancer, prostate cancer, hormone-driven cancers, SN-38, immunotherapy

## Abstract

Breast and prostate cancers, two of the most prevalent malignancies worldwide, pose significant therapeutic challenges owing to their resistance to conventional treatments and complex tumor microenvironments. The integration of innovative therapies into current clinical frameworks is essential for improving patient outcomes. SN-38, an active metabolite of irinotecan, exerts potent antitumor effects by inhibiting topoisomerase I and modulating the tumor microenvironment. In addition to direct cytotoxicity, SN-38 induces immunogenic cell death, promotes damage-associated molecular pattern (DAMP) release, and enhances antitumor immune responses. These dual mechanisms support the potential of combining it with chemotherapy, targeted therapy, and immunotherapy, particularly in breast and prostate cancers. However, challenges such as poor solubility, rapid degradation, and dose-limiting toxicity hinder its clinical translation. Novel delivery systems, including liposomal formulations, antibody–drug conjugates, and nanoparticle-based strategies, are being developed to address these limitations. This review summarizes the current evidence on SN-38 alone and in combination with emerging therapies, highlighting its potential as a dual cytotoxic and immune-modulating agent in resistant and aggressive cancers.

## 1. Introduction

Hormone-driven cancers, such as prostate and breast cancers, are otherwise known as hormone-dependent or hormone-sensitive cancers, and they account for a higher significant of cancer diagnoses worldwide [1,2,3,4], highlighting the critical need for effective therapeutic interventions. Breast cancer is the most commonly diagnosed malignancy worldwide, with an estimated 2.3 million new cases in 2020 alone [1], while prostate cancer (PCa) is a significant health burden, ranking as one of the leading causes of cancer-related death in men worldwide [3,4]. Both cancers rely on estrogen receptor (ER) and androgen receptor (AR) signaling, for their initiation and progression, emphasizing the need for advanced therapeutic strategies in disease treatment.

Despite the substantial progress in the therapeutic strategies in cancer research, the management of hormone-driven cancers remains one of the major clinical challenges. Endocrine therapies are highly effective in the initial stage of hormone-driven cancers treatment by targeting hormone receptor pathways; nevertheless, the development of resistance overtime has greatly impeded their long-term positive outcome, which is a key hindrance to effective cancer management [5,6]. In addition, the heterogeneity nature of these two cancers causes inconsistency in the treatment responses and promotes the development of therapy-resistant clones [7,8,9]. Thus, this complexity stresses the crucial need for novel therapeutic approaches that can improve treatment efficacy and tackle resistance challenges.

SN-38, also called 7-ethyl-10-hydroxycamptothecin, is a significantly potent active metabolite of irinotecan that has shown promise in oncology treatment [10]. SN-38 exerts its anti-cancer activities by binding to and inhibits topoisomerase I, an essential enzyme for DNA replication by stabilizing the cleavable complex between topoisomerase I and DNA, which cause DNA breaks, inhibition of DNA replication through cell cycle arrest, and apoptosis [11,12]. It has been documented that SN-38 has anti-cancer activity against many types of cancer, this includes colorectal, small cell lung, lymphoma, breast, esophageal, uterine, and ovarian cancers [13], this has underscored its useful option as a versatile chemotherapeutic agent.

The clinical application of SN-38 as a great potential for the treatment of cancer has been hampered primarily by its poor water solubility and instability caused by spontaneous hydrolysis as well as systemic toxicities, which avert its direct administration as a drug [14]. Recently, researchers have focused on improving the bioavailability and adverse effects of SN-38 using advanced drug delivery systems technology [13,15,16]. Even with these challenges, SN-38 is still an interesting drug molecule because of its ability to directly target tumor cells, which could enhance its therapeutic efficacy and potential when combined with other cancer treatments. Nonetheless, its promise in combination therapy aimed at hormone-driven malignancies remains little investigated.

In cancer treatment, immunotherapy has transformed the treatment approach through the use of the body immune system to recognize and eliminate cancerous cells [17]. Recent immunotherapy treatments called immune checkpoint inhibitors, like anti-PD-1/PD-L1 and anti-CTLA-4 antibodies, have shown great results in fighting several cancers, by reactivating and rejuvenating worn-out T cells and boosting the anti-tumor immunity to fight tumors [18,19]. The additional modalities, such as cancer vaccines, oncolytic viruses, and adoptive T cell therapies continue to broaden the therapeutic landscape. However, in hormone-driven cancers like breast and prostate cancers, immunotherapy faces unique challenges. For instance, the immunosuppressive tumor microenvironment (TME) associated with these cancers often inhibits immune cell infiltration and function, thereby limiting the efficacy of immune-based interventions [20,21]. Furthermore, the interplay between hormone receptor signaling and immune evasion mechanisms creates additional barriers to successful immunotherapy. Thus, overcoming these obstacles requires a nuanced approach that combines immunotherapy with agents like SN-38 to modulate the TME and enhance immune system engagement. This review is intended as a mechanistic overview, focusing on the fundamental biological principles underlying SN-38 activity and its potential synergy with immunotherapy. While several preclinical studies are cited, our emphasis is on the mechanistic insights they provide rather than on exhaustive methodological details such as dosage, timing, or experimental model design.

## 2. SN-38: Mechanism of Action and Current Applications

SN-38, the active metabolite of irinotecan, is a potent topoisomerase I inhibitor that stabilizes the cleavable complex between the enzyme and DNA, leading to the accumulation of DNA strand breaks, replication stress, and subsequent apoptosis [22,23]. In addition to its cytotoxic activity, SN-38 has been shown to induce immunogenic cell death (ICD), thereby contributing to antitumor immune responses. Owing to these properties, SN-38 has been widely studied in breast and prostate cancers, both as a monotherapy and in combination with other therapeutic agents. However, the pharmacokinetics of SN-38 is subject to significant inter-individual variability, which is influenced by both genetic and environmental factors. A key determinant of SN-38 exposure is the activity of UDP-glucuronosyltransferase 1A1 (UGT1A1), which catalysis the glucuronidation of SN-38 to its inactive form, SN-38G [23,24]. Genetic polymorphisms in *UGT1A1*, particularly UGT1A1*28 and UGT1A1*6, have been associated with impaired SN-38 detoxification and increased risk of toxicity [24,25].

### Applications of SN-38 in Oncology

The application of SN-38 is pivotal in oncology because of its potent antitumor properties, particularly in the treatment of various cancers, such as metastatic colorectal cancer and glioblastoma. SN-38 functions by inhibiting topoisomerase I, an enzyme crucial for DNA replication, thereby inducing double-stranded DNA breaks and eventual cell death during the mitotic S-phase of cancer cells [26]. Its efficacy in colorectal cancer has been notably enhanced through combination therapies, including the use of antibody-drug conjugates (ADCs), such as sacituzumab govitecan, which targets the TROP-2 antigen expressed in several cancer types [27]. This ADC not only stabilizes SN-38 but also enhances its delivery to cancer cells while mitigating the side effects, thus improving the therapeutic outcomes.

In glioblastoma, SN-38 has demonstrated superior antitumor activity compared to its prodrug irinotecan (CPT-11), suggesting its effectiveness against resistant and multidrug-resistant glioma cells [28]. Moreover, the incorporation of SN-38 with PARP inhibitors, such as olaparib, demonstrates a synergistic antitumor effect by intensifying DNA damage and disrupting repair pathways in cancer cells, further expanding its application in combination therapies [29]. Furthermore, innovative approaches, such as bio-orthogonal uncaging using palladium-functionalized devices, have been employed to control the release and activation of SN-38 at tumor sites, thereby reducing systemic toxicity and enhancing therapeutic indices [26]. The pharmacogenetics of SN-38 metabolism also play a crucial role; polymorphisms in UGT1A1 that impair the inactivation of SN-38 can lead to increased drug exposure and toxicity, necessitating dose individualization strategies (personalized treatments) for improved patient outcomes [23,30].

SN-38 has also showed significant promise in oncology, particularly when combined with immunotherapy approaches for breast and prostate cancers. This promise is not only theoretical but also supported by emerging clinical applications and research [10]. In breast cancer, particularly in challenging subtypes such as triple-negative breast cancer (TNBC), SN-38 can be combined with immunotherapeutic strategies to enhance treatment efficacy. TNBC is characterized by the absence of estrogen and progesterone receptors and HER2 protein, making it less responsive to conventional hormone therapy. However, TNBC shows a higher tumor mutation burden and increased presence of tumor-infiltrating lymphocytes, which primes it for a response to immunotherapy [31]. Current immunotherapeutic advancements focus on immune checkpoint inhibitors, such as pembrolizumab, combined with chemotherapeutic agents, which have shown efficacy in TNBC [32]. The potential integration of SN-38 with these immunotherapeutic approaches could enhance antitumor activity, offering a more robust treatment regimen that leverages both the cytotoxic potential of SN-38 and the immune-modulating capabilities of the checkpoint inhibitors.

Similar challenges exist in prostate cancer because of its characterization as an immunologically ‘cold’ tumor with intrinsic resistance to immune checkpoint inhibitors. Nonetheless, the combination of SN-38, which exerts cytotoxic effects through topoisomerase I inhibition, with immunotherapeutic agents offers the potential for better clinical outcomes and is a crucial area of investigation [33]. Combination strategies have shown promise in revamping the immunosuppressive environment typical of prostate tumors, particularly when combined with treatments such as vaccine-based therapies or drugs targeting the immunosuppressive TME [34,35]. By utilizing SN-38 ability to induce DNA damage along with immunomodulation to decrease immune evasion, there is an opportunity to augment anti-tumor immune responses, potentially leading to improved survival rates. Moreover, ongoing efforts in clinical trials need to seek to optimize these synergies by identifying the most effective biomarkers for treatment stratification and monitoring of treatment responses [36]. The exploration of SN-38 role in combination with other immunotherapy modalities, such as PD-1/PD-L1 inhibitors, continues to evolve, signaling new directions that could redefine the therapeutic landscape in these cancer types [37]. In summary, SN-38 holds significant potential in oncology, with expanding applications in breast and prostate cancers. Its integration with immunotherapy represents a frontier of innovative treatment paradigms aimed at reducing mortality and improving the quality of life of patients with cancer.

## 3. Immunotherapy in Breast and Prostate Cancer

### 3.1. Advances in Immunotherapy

Immunotherapy has emerged as a promising approach for the treatment of breast and prostate cancers, despite the initial challenges in harnessing its full potential. In breast cancer, which was historically considered non-immunogenic, recent studies have revealed that a subset of tumors exhibits immune activation and infiltration through tumor-infiltrating lymphocytes (TILs), particularly in TNBC [38]. The approval of pembrolizumab in combination with chemotherapy for PD-L1-positive metastatic TNBC marks a significant milestone, demonstrating improved progression-free survival [32,38].

In prostate cancer treatment, the combination of low immunogenicity, specific genetic alterations, immunosuppressive TME, and impaired cellular immunity creates a multifaceted barrier to effective immunotherapy in prostate cancer, which is characterized by low levels of antigen presentation, limited cytotoxic T-cell activation, and high expression of immune checkpoint molecules and immunosuppressive cytokines/chemokines [39]. TME plays a crucial role in suppressing antitumor immune responses through complex interactions between tumor cells, stromal cells, and immune cells [40]. Specific molecular alterations contribute to immune evasion in prostate cancer. Somatic mutations in genes such as *PTEN, TP53, RB1, CDK12*, and DNA repair genes, as well as the activation of pathways such as ETS and MYC, can facilitate immune evasion [41]. Additionally, the presence of immunosuppressive cells, such as myeloid-derived suppressor cells and tumor-associated macrophages, in the TME further compromises immune responses [40]. The low tumor mutational burden in prostate cancer is another factor limiting immunotherapy efficacy [42]. This results in fewer neoantigens for the immune system to recognize and target.

Furthermore, impaired cellular immunity and recruitment of immunosuppressive cells contribute to the overall immunosuppressive environment [39,42]. Overcoming these resistance mechanisms is crucial for improving treatment outcomes, and current research focuses on combination strategies that target multiple aspects of immune evasion simultaneously [42]. Despite the limited success of immunotherapy in prostate cancer, its treatment and management have seen renewed interest due to ongoing research into novel therapeutic targets and combination strategies [33,35]. The identification of key mechanisms of immune resistance in the prostate TME has led to the discovery of new treatment targets, which are currently being translated into innovative clinical trials [33].

Both cancer types are currently exploring combination approaches to enhance the efficacy of immunotherapy. These include dual immune checkpoint inhibition, bispecific antibodies and novel ADCs [32,38]. Strategies that combine immunotherapy with standard treatments, such as chemotherapy, targeted therapy, and radiotherapy, are being investigated [43]. The potential of nanotechnology to improve the delivery of immunotherapeutics to the TME is also being explored [38].

Despite these advancements, several challenges remain. Only a subset of breast cancers responds to current immunotherapies, and prostate cancer continues to show limited success [33,38]. Ongoing research has focused on overcoming these limitations through personalized approaches and development of predictive biomarkers [44,45]. In addition, overcoming prostate cancer resistance mechanisms to immunotherapy is crucial to improve treatment outcomes, and current research focuses on combination strategies to simultaneously target multiple aspects of immune evasion [42]. As our understanding of tumor immunology deepens, targeted and personalized immunotherapy is likely to become an integral part of cancer care, especially when used in combination with complementary treatment strategies for both breast and prostate cancers.

### 3.2. Challenges in Hormone-Driven Cancers

Hormone-driven cancers, such as breast and prostate cancers, present unique challenges in immunotherapy. In breast cancer, the hormone receptor-positive (HR+) subtype, which accounts for the majority of cases, has shown a limited response to immunotherapy compared to TNBC [36]. This is partly due to the immunologically “cold” nature of HR+ tumors, characterized by low levels of tumor-infiltrating lymphocytes and a less immunogenic microenvironment [36,46]. Similarly, prostate cancer, which is initially androgen-dependent, poses significant hurdles to immunotherapy. As the disease progresses to castration-resistant prostate cancer (CRPC), it becomes increasingly difficult to treat with current therapies including immunotherapy [47]. The complex TME of prostate cancer, with its various immunosuppressive mechanisms and low tumor mutational burden, contributes to its classification as a “cold” tumor, limiting the efficacy of immune checkpoint inhibitors [33,47].

Despite these challenges, the advancements in understanding the molecular mechanisms of immune evasion and the development of novel approaches, such as combination therapies and targeted delivery systems, offer promising avenues for improving immunotherapy outcomes in hormone-driven cancers [44,48]. For instance, the integration of combination immunotherapy for breast and prostate cancers is rapidly evolving. Researchers are exploring diverse strategies including chemoimmunotherapy, nanoparticle-based delivery systems, and combinations of vaccines, checkpoint inhibitors, and targeted therapies. These approaches aim to overcome the limitations of monotherapy and improve clinical outcomes in patients with challenging cancers.

Novel combination therapies are being extensively explored to enhance immunotherapy outcomes in breast and prostate cancers, addressing the limitations of monotherapy. In breast cancer, particularly metastatic triple-negative breast cancer (mTNBC), several immune-based combinations have been investigated to improve overall response and clinical outcomes [49]. Chemoimmunotherapy has shown notable results and has been approved for PD-L1 positive mTNBC patients. Numerous trials are exploring novel immune checkpoint inhibitor (ICI)-based combinations, with anticipated results [49]. Additionally, nanotechnology is being integrated with immunotherapy to maximize its efficiency and reduce toxic side effects. Nanoparticles are being used for direct activation of immune systems through delivery of tumor antigens and adjuvants, altering immunosuppression of the tumor environment, and in combination with conventional therapies [50]. For prostate cancer, which has shown a limited response to immunotherapy alone, various combination strategies are being explored. These include combining ICIs with other treatments to reduce drug resistance and attack cancer cells through multiple cellular pathways [51]. Novel approaches include the combination of immunotherapy with chemotherapy, targeted therapy, vaccines, and radiation [51]. Specific combinations being investigated are cancer vaccines with immune checkpoint blockade, which simulations predict as potentially the most effective dual-drug combination for androgen deprivation therapy-resistant subjects [52]. Additionally, PARP inhibitors, such as olaparib, have shown promising results in combination with standard treatments for metastatic castration-resistant prostate cancer patients with DNA repair defects [53].

The integration of nanomedicine with immunotherapy has shown the potential to enhance treatment efficacy and overcome the immunosuppressive TME in prostate cancer [48,54]. Additionally, emerging strategies like bispecific T-cell engagers (BiTEs) and personalized approaches based on molecular subtyping and genetic profiling may help address the limitations of current immunotherapies in these challenging cancer types [55,56].

### 3.3. Opportunities for Combination Therapies

The evolving landscape of immunotherapy for breast and prostate cancers presents promising avenues for combination therapies. The combination of immune checkpoint inhibitors with chemotherapy has shown encouraging results in breast cancer, particularly in TNBC [57]. The approval of pembrolizumab in combination with chemotherapy for PD-L1 positive metastatic and early-stage TNBC is a significant milestone [32]. This success has paved the way for exploring similar combinations in other breast cancer subtypes, including hormone receptor-positive and HER2-positive diseases.

While immunotherapy as a monotherapy has shown limited efficacy for prostate cancer, combination approaches are being actively investigated to enhance treatment outcomes [35]. Ongoing trials are exploring immune checkpoint inhibitors in combination with various agents, including androgen axis inhibitors, PARP inhibitors, radium-223, radiotherapy, cryotherapy, tumor vaccines, chemotherapy, and tyrosine kinase inhibitors [35,58]. These combinations aim to modulate the immune system and overcome biological barriers that have historically limited the success of immunotherapy in prostate cancer.

Beyond the standard checkpoint blockade, innovative combinations are being explored for both cancer types. In breast cancer, ADCs are paired with checkpoint inhibitors, and emerging research is evaluating bispecific antibodies, oncolytic viruses, and therapeutic cancer vaccines as part of multipronged strategies [32]. In prostate cancer, advanced approaches include targeting the adenosine signaling axis, utilizing bispecific T-cell engagers, PSMA-directed therapies, and personalized adoptive T-cell therapies, such as CAR-T cells [35,59].

Synergistic effects are being pursued by combining multiple immunotherapies. For example, modeling studies have suggested that pairing cancer vaccines with immune checkpoint blockade may offer substantial benefits in androgen-deprivation-resistant prostate cancer [52]. Similarly, combining engineered immune cells, such as CAR-Ts, with checkpoint inhibition and tumor vaccines may amplify immune responses in breast cancer, especially in hard-to-treat cases [44]. Moreover, strategies that modify the TME are gaining traction. Nucleic acid-mediated immune stimulation is being investigated to enhance checkpoint inhibitor efficacy [60], and anti-angiogenic therapies have shown promise in improving immune infiltration and normalizing the tumor vasculature to support immunotherapy responsiveness [61].

In summary, the future of immunotherapy in breast and prostate cancers depends on the thoughtful integration of combination therapies. These approaches aim not only to activate immune responses but also to dismantle barriers within the TME. As research progresses, the identification of predictive biomarkers and optimization of treatment sequences will be essential for tailoring these therapies to individual patient needs, ultimately improving clinical outcomes in these complex and historically resistant cancers [14,59,62].

## 4. Synergy Between SN-38 and Immunotherapy

**Figure 1:**
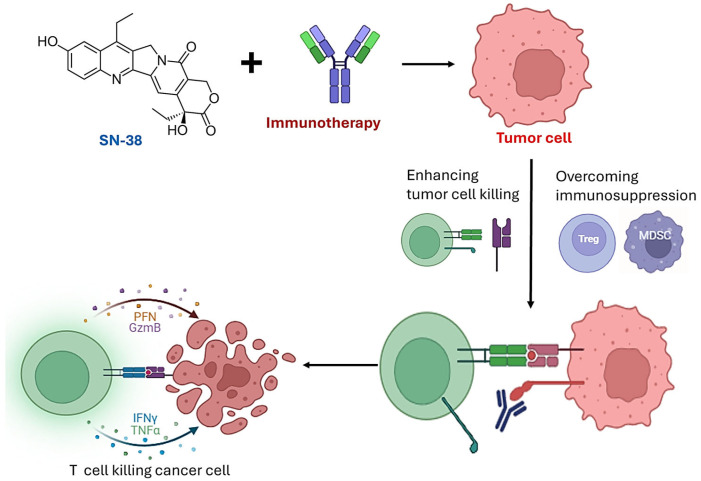
Synergistic potential of SN-38 and immunotherapy in tumor eradication. SN-38 induces tumor cell death through DNA damage and apoptosis. Beyond direct cytotoxicity, SN-38 can generate immunogenic cell death (ICD), releasing tumor antigens and danger-associated molecular patterns (DAMPs), which prime an immune response. The immune checkpoint inhibitor counteracts tumor-induced immunosuppression via Tregs, MDSCs, PD-1/PD-L1 interaction. This restores T-cell cytoxic activity against tumor cells.

Drug combinations are commonly used to treat cancers, manage pain, combat infections, and address a variety of other medical conditions. The concept of synergism in pharmacology occurs when two or more drugs work together in a way that produces a combined effect higher than the sum of their individual effects [63]. Rather than simply adding their benefits, the drugs amplify each other’s actions, achieving a stronger therapeutic outcome. Understanding and evaluating synergism requires rigorous analysis to confirm that the combination produces enhanced outcomes [63]. One of the key advantages of synergistic drug interactions is the ability to lower the required dosages of each drug, which can help reduce adverse effects and improve patient tolerability [63,64]. This principle is particularly valuable in cancer treatment, where high-dose chemotherapy often leads to significant toxicity. By leveraging synergy, it is possible to enhance therapeutic outcomes while minimizing harm to normal tissues. Thus, this section discussed the synergistic combinatory effects between SN-38 and immunotherapy agents in cancer treatment. As shown in Figure 1, the combination of SN-38 with immunotherapy may provide a complementary strategy for cancer treatment. SN-38 induces direct cytotoxicity through DNA damage, leading to tumor cell apoptosis and increased release of tumor-associated antigens. These antigens can prime and enhance T-cell activation, making the tumor more susceptible to immune recognition. Meanwhile, immunotherapy reverses immune evasion by blocking checkpoint pathways, counteracting Treg and MDSC-mediated suppression, and enabling T cells to exert robust cytotoxic responses. Activated T cells kill tumor cells through perforin- and granzyme-dependent apoptosis as well as cytokine secretion (IFNγ, TNFα). Together, this combination could enhance tumor eradication, reduce the likelihood of resistance, and support durable anti-tumor immunity.

### 4.1. Immunogenic Effects of SN-38

Beyond the cytotoxicity effects of SN-38, it has been reported for its immunogenic potential, which makes it a promising agent in combination with immunotherapy. By inducing immunogenic cell death (ICD), SN-38 facilitates the release of damage-associated molecular patterns (DAMPs) such as ATP and high mobility group box 1 (HMGB1) [65]. Gong and co-workers [65] created a special hydrogel that responds to reactive oxygen species (ROS). This hydrogel combines SN38 and anti-PDL1 antibodies for localized cancer therapy. This system leverages ROS-triggered hydrogel degradation to release SN38, inducing immunogenic cell death, and aPDL1, boosting T cell responses, effectively suppressing tumor growth. Additionally, it promotes calreticulin exposure on the cell surface [66]. These signals enhance dendritic cell activation and antigen presentation, effectively priming T-cell responses against tumor-associated antigens [66]. Research highlights the combinatorial potential of SN-38 with other agents. For instance, Jiang et al. [66] demonstrated that the combination of oxaliplatin and SN-38 enhances cytotoxicity by not only inhibiting DNA replication but also triggering ICD. This process leads to increased PD-L1 expression on both tumor cells and dendritic cells (DCs), thereby promoting immune system activation and strengthening antitumor responses. The elevated PD-L1 levels facilitate cytotoxic T lymphocyte (CTL) proliferation and tumor infiltration, enhancing their ability to destroy cancer cells. Additionally, the upregulation of PD-L1 makes tumor cells more susceptible to PD-L1 blockade therapy, further reinvigorating T cell activity and amplifying immune-mediated tumor eradication. Moreso, the expression of calreticulin (CRT) on the surface of tumor cell triggers the release of DAMPs, such as HMGB1 and ATP, signals which could further enable antigen-presenting cells (APCs) to phagocytes dying tumor cells and present tumor antigens [66]. Moreover, the delivery of SN-38 via advanced formulations, such as core–shell nanoparticles, enhances its tumor deposition and antitumor efficacy. This approach optimizes the synergy between SN-38 and immune checkpoint blockade therapies, demonstrating significant tumor regression in preclinical models. Collectively, these findings underscore the dual role of SN-38 in directly targeting cancer cells and modulating the immune system to achieve superior therapeutic outcomes [66].

A low dose of SN-38, with minimal cytotoxic effects, significantly downregulated PD-L1 expression in the ovarian cancer cell line OVCA429 and the breast cancer cell line MCF-7. Furthermore, SN-38 exhibits considerable antitumor effects in ovarian and breast cancers by modulating natural killer (NK) cell activity [67]. In head and neck squamous cell carcinoma (HNSCC), SN-38 significantly reduces PD-L1 expression, which in turn inhibits the PD-L1/PD-1 checkpoint, promoting tumor suppression or elimination. Using low doses of targeted therapies alongside immunotherapy can modulate immune cells without the severe side effects associated with higher doses [68]. In a mouse breast tumor model, the combination of anti-PD-L1 antibodies with irinotecan, a prodrug of SN-38, has shown promise for improving cancer treatment outcomes [69]. Research on the FM3A murine tumor model demonstrated that while irinotecan initially caused a temporary reduction in peripheral blood lymphocytes, its combination with anti-PD-L1 therapy produced significantly greater antitumor effects than either agent alone. This enhanced efficacy was linked to an increase in CD8+ T cell proliferation within both tumors and lymph nodes, leading to a higher presence of tumor-infiltrating CD8+ cells compared to monotherapy. Additionally, irinotecan reduced the number of regulatory T cells (Tregs) in tumors and lymph nodes, further promoting CD8+ cell expansion, and this was also observed when Tregs were selectively depleted using anti-folate receptor 4 antibodies [69]. Moreover, irinotecan upregulated MHC class I expression on tumor cells, improving antigen presentation while simultaneously increasing PD-L1 expression on both tumor cells and immune cells within the TME. The elevated PD-L1 levels were effectively countered by the anti-PD-L1 antibody, ensuring sustained immune activation [69]. These suggest that irinotecan enhances T cell activation in anti-PD-L1 therapy by reducing immune suppression through Treg depletion and strengthening tumor antigen presentation via MHC class I upregulation.

### 4.2. Modulation of the Tumor Microenvironment

The immunosuppressive nature of the TME poses a significant barrier to effective cancer immunotherapy [70]. SN-38 can remodel the TME by altering the cellular and molecular landscape in favor of antitumor immunity [66,67]. SN-38 has been shown to suppress acute inflammatory response by obstructing lipopolysaccharide (LPS)-induced toll-like receptor 4 activation in macrophages [71]. In TME, the interaction between cancer cells and immune cells, such as macrophages, frequently induces a chronic inflammatory response via TLR4 activation, facilitated by the release of danger signals like LPS, resulting in the secretion of pro-inflammatory cytokines that enhance tumor growth and metastasis [72,73]. Thus, inhibiting the interaction between LPS and TLR4, or obstructing the subsequent signaling cascade, can limit macrophage activation and the production of inflammatory mediators, hence enhancing the immune system ability to identify and target cancer cells, which improves anti-tumor immune responses [72,73]. Interestingly, topoisomerase-1 inhibitors have been demonstrated to suppress inflammatory genes and protect animals against LPS-induced mortality by modulating RNA polymerase II activity [74].

Simultaneously, SN-38 enhances the infiltration of cytotoxic CD8+ T cells and natural killer (NK) cells into the TME [67]. These immune cells alter the TME by secreting cytokines that promote antitumor immune responses while suppressing tumor-supporting factors [67,68,75]. Chung and colleagues [67] demonstrated that SN-38 could act as a potent modulator of the TME through its capacity to suppress PD-L1 expression. Mechanistically, SN-38 achieves this by downregulating c-Myc and STAT3, key regulators of PD-L1, while concurrently promoting FOXO3 activation, which is essential for this suppression. c-Myc is known to enhance the production of immunosuppressive factors while repressing immune activation regulators, thereby facilitating immune evasion in tumors [76]. Similarly, STAT3, apart from its oncogenic role in modulating gene expression, promotes cancer progression through immunosuppression. STAT3 activation in immune cells suppresses immune mediators and enhances immunosuppressive factors within the TME [77,78]. Chung et al. [67] further reported that synergistic effects of SN-38 with metformin enhance antitumor immunity by facilitating the infiltration of NK and CD8+ T cells into the TME, leading to increased secretion of interferon-γ and granzyme B, critical mediators of tumor cell killing. Furthermore, SN-38 sensitizes unresponsive tumors to anti-PD-1 therapy, highlighting its potential in overcoming resistance to immunotherapy [67]. These findings underscore the therapeutic promise of SN-38 in reprogramming the TME to bolster patient responses to immunotherapy.

### 4.3. Overcoming Resistance Mechanisms

Resistance to immunotherapy remains a significant challenge in cancer treatment, often arising from mechanisms such as T-cell exhaustion, inadequate antigen presentation, and adaptive immune resistance. Emerging evidence suggests that SN-38 can help overcome these resistance pathways, thereby enhancing the efficacy of immunotherapy. One key mechanism of tumor resistance is the downregulation of antigen-processing machinery (APM) components, including MHC class I molecules, which impairs immune recognition and tumor elimination [79,80]. Studies have demonstrated that SN-38 can restore APM functionality, leading to improved antigen presentation and heightened sensitivity to immune-mediated destruction [81]. Liang et al. [81] reported that SN-38 upregulates stimulatory MHC class I alleles by activating TAP1 and TAP2, thereby enhancing antigen presentation in cancer cells. Additionally, SN-38 has been shown to promote the phagocytosis of colon cancer cells by monocyte-derived dendritic cells (MoDCs), further supporting its role in enhancing immune recognition.

Within the TME, SN-38, in combination with metformin, has been shown to enhance antitumor immunity by inhibiting c-Myc and STAT3 via FOXO3 activation [67]. MYC, a transcription factor essential for cell proliferation, is frequently dysregulated in aggressive tumors and has been identified as a resistance factor to ICIs. Recent studies highlight MYC overexpression as a potential biomarker and therapeutic target in recurrent and metastatic head and neck squamous cell carcinoma (HNSCC) [82,83]. Similarly, aberrant STAT3 signaling contributes to carcinogenesis and resistance to both chemotherapy and targeted therapies [84]. RNA sequencing analyses in HNSCC suggest that SN-38 modulates the immune microenvironment by promoting immune cell infiltration and upregulating immune-related genes, further supporting its potential role in enhancing the efficacy of immunotherapy [68]. Another critical resistance mechanism involves the presence of an immunosuppressive TME [85,86], which SN-38 counteracts by recruiting and activating effector immune cells [67]. Additionally, SN-38 may inhibit DNA damage repair pathways in tumor cells, increasing genomic instability and promoting the generation of neoantigens [87,88,89]. These neoantigens serve as novel immune targets, expanding the therapeutic potential of immunotherapy in resistant cancers [90].

## 5. Therapeutic Potential, Obstacles, and Future Perspectives

### 5.1. Emerging Preclinical Evidence of SN-38 Synergy with Immunotherapy

Preclinical research has unveiled SN-38 as a promising agent for reshaping the TME in breast and prostate cancer. The synergy between SN-38 and immunotherapy is supported by its ability to promote the activation of natural killer (NK) cells and CD8+ T cells, leading to the increased secretion of IFN-γ and granzyme B, both of which are critical for tumor cell destruction [67,68]. Several studies suggest that SN-38-mediated modulation of the TME enhances immune infiltration and tumor suppression [67,68,91]. Mechanistically, SN-38 exerts its effects by interfering with key oncogenic signaling pathways such as the STAT3/IL-6 axis, c-Myc regulation, and PI3K/AKT/mTOR signaling [91]. By inhibiting STAT3 phosphorylation and reducing IL-6 levels, SN-38 prevents the upregulation of PD-L1, thereby strengthening immune recognition of tumor cells [67,91]. Similarly, SN-38 suppresses c-Myc expression, which is associated with tumor progression and immune evasion. Through these molecular alterations, SN-38 creates a more immunogenic TME that is responsive to immunotherapy [91]. Preclinical studies, including in vitro experiments and in vivo mouse tumor models, have demonstrated that low doses of SN-38 significantly suppress tumor growth. Interestingly, even at non-toxic doses, SN-38 induces a potent immune response by recruiting NK cells into the TME and enhancing their cytotoxic activity [91]. These findings highlight the potential of SN-38 as an immune-activating agent that could be integrated into clinical settings to improve the efficacy of ICIs.

Gong and co-workers [65] present a reactive oxygen species (ROS)-responsive hydrogel, designed for localized delivery of anti-PD-L1 (aPDL1) antibodies. The hydrogel, formulated by cross-linking SN38-SA–BA with poly(vinyl alcohol) (PVA), degrades in the presence of ROS, releasing free SN38 and encapsulated aPDL1. SN38 induces immunogenic cell death (ICD), triggering the release of damage-associated molecular patterns (DAMPs) that stimulate the immune system, while aPDL1 blocks PD-L1 on cancer cells, enhancing T cell-mediated antitumor immunity. This dual-action system effectively promotes both innate and adaptive immune responses, leading to significant tumor suppression and potential eradication [65]. By integrating SN-38 with immunotherapy, this biomaterial-based approach offers a promising strategy to enhance cancer treatment efficacy and overcome immune resistance.

The synergy between SN-38 and immunotherapy represents a transformative approach in cancer treatment, addressing therapeutic resistance and improving survival outcomes in preclinical models. Future clinical trials should focus on optimizing SN-38 dosing strategies and identifying biomarkers that predict patient response to this combination therapy. Overall, SN-38 represents a promising therapeutic approach for overcoming immunotherapy resistance in breast and prostate cancers by simultaneously targeting tumor survival pathways and enhancing immune-mediated tumor destruction.

### 5.2. Clinical and Formulation Limitations of SN-38

Despite its potent antitumor activity, the clinical translation of SN-38 is hampered by several pharmacological and formulation challenges. A major limitation is its extremely poor aqueous solubility, which restricts oral and intravenous administration and results in highly variable systemic exposure. In aqueous environments, SN-38 is also prone to rapid degradation and hydrolysis, further limiting its stability and therapeutic window.

Metabolism via uridine diphosphate glucuronosyltransferase (UGT1A1) contributes to rapid clearance and interpatient variability. Genetic polymorphisms in *UGT1A1* can result in excessive accumulation of SN-38, leading to increased risk of dose-limiting toxicities such as severe diarrhea and myelosuppression [25]. These toxicities significantly complicate dosing strategies and have restricted broader clinical use of SN-38 as a free drug.

In addition to pharmacokinetic issues, the physicochemical instability of SN-38 has limited the development of conventional formulations. Its high lipophilicity, combined with sensitivity to environmental conditions, contributes to poor reproducibility of drug release and suboptimal bioavailability [38]. Collectively, these challenges highlight the need for innovative delivery systems and formulation approaches to harness the therapeutic potential of SN-38 while minimizing its liabilities. To overcome these limitations, advanced drug delivery systems such as liposomal formulations, polymeric nanoparticles, and ADCs have been developed. These nanotechnology-based strategies enhance tumor-specific drug accumulation, improve bioavailability, and minimize off-target toxicity, thereby optimizing SN-38’s therapeutic potential. Koliqi et al. [92] explored PEO-PPO-PEO/P(DL)LCL nanoparticles as a delivery system for SN-38, improving drug solubility and stability while maintaining high encapsulation efficiency. The study emphasized the importance of surface modifications in enhancing tumor targeting and reducing toxicity, highlighting the potential of these nanoparticles for SN-38-based cancer therapy. Similarly, Mehdizadeh et al. [93] developed biotin-decorated PLGA nanoparticles, leveraging biotin receptor overexpression in cancer cells to improve SN-38 uptake, prolong drug release, and enhance cytotoxicity while minimizing systemic toxicity.

Targeted drug delivery has also been applied to neuroblastoma therapy, as demonstrated by Monterrubio et al. [94]. Their study developed anti-GD2 antibody-functionalized SN-38 nanoparticles, which exhibited superior tumor penetration and retention, leading to enhanced survival in patient-derived xenograft (PDX) models. This approach improved drug localization and therapeutic efficacy while reducing systemic toxicity. In contrast, Narsinh et al. [95] explored convection-enhanced delivery (CED) of liposomal irinotecan in glioblastoma patients, utilizing real-time MRI guidance to optimize tumor coverage. This strategy improved drug distribution while limiting systemic toxicity, though further clinical studies are needed to assess its full therapeutic potential.

To overcome chemoresistance in colorectal cancer, Huang et al. [96] designed BI@PEG-SN38 nanoparticles, which co-deliver SN-38 and a BCRP inhibitor (Ko143). These nanoparticles exhibited high drug-loading efficiency, selective tumor release, and improved therapeutic efficacy in resistant cancer cells. Preclinical models have demonstrated that combining SN-38 with immunotherapeutic agents, including ICIs and SN-38-based ADCs, enhances therapeutic efficacy compared to monotherapy. Sharkey et al. [97] investigated SN-38 ADCs targeting CD22 (epratuzumab) and CD20 (veltuzumab) in B-cell malignancies. While both conjugates exhibited potent antitumor activity, the rapid internalization of epratuzumab-SN-38 led to superior efficacy, despite the lower CD22 expression. In vivo, epratuzumab-SN-38 effectively eradicated tumors at nontoxic doses and exhibited enhanced potency when combined with veltuzumab, highlighting its potential in combination therapy. Additionally, SN-38 has been successfully conjugated to a humanized antibody against trophoblast cell surface antigen 2 (TROP-2), a key regulator of cancer signaling pathways that is overexpressed in multiple malignancies [27]. This approach led to the development of sacituzumab govitecan, an ADC designed to improve targeted drug delivery in cancer therapy [27,98]. Similarly, Yang et al. [99] developed SN-38-loaded human serum albumin (HSA) and hyaluronic acid (HA) nanoparticles (SH/HA NPs) for chemo-radiotherapy in CD44-expressing cancers. These nanoparticles enhanced radiosensitization, promoted G2/M phase cell cycle arrest, and improved tumor suppression in vivo, offering a promising approach to improving chemo-radiotherapy efficacy while minimizing side effects. Further advancements in controlled drug release have been achieved by Jiang et al. [66], who developed a core–shell nanoparticle (OxPt/SN38) for two-stage SN-38 release. This system ensures controlled esterase-mediated release in the liver and acid-triggered hydrolysis in tumors, significantly improving tumor-specific drug accumulation. Additionally, the formulation demonstrated synergy with immune checkpoint inhibitors, upregulating PD-L1 expression, promoting immunogenic cell death, and enhancing T-cell infiltration, positioning OxPt/SN38 as a promising candidate for combination immunotherapy.

These collective findings highlight the transformative potential of nanotechnology-driven SN-38 delivery systems. By enhancing solubility, improving tumor targeting, and overcoming resistance mechanisms, these innovations pave the way for more effective and less toxic cancer treatments. Future research should focus on clinical translation, biomarker-driven patient selection, and combination strategies to fully harness SN-38’s therapeutic potential in cancer therapy.

### 5.3. Biomarker-Driven Strategies for Optimizing SN-38 and Immunotherapy Combinations

Biomarker-driven patient stratification has become a critical approach in optimizing the efficacy of immunotherapy and targeted therapies like SN-38 in breast and prostate cancers. Given the heterogeneity of these malignancies, the identification of reliable biomarkers is essential for guiding treatment decisions, predicting patient responses, and improving clinical outcomes. By integrating genomic, proteomic, and immune-related biomarkers, precision medicine can enhance therapeutic efficacy while minimizing toxicity. In breast cancer, PD-L1 expression is widely recognized as a predictive biomarker for ICIs [45]. However, its limitations highlight the need for alternative biomarkers. Recent advances have identified immune gene signatures, tumor-infiltrating lymphocytes (TILs), and c-Myc activity as potential indicators of treatment responsiveness [100]. Similarly, in prostate cancer, biomarkers beyond prostate-specific antigen (PSA) are being explored. The 4Kscore test and Prostate Health Index have demonstrated efficacy in distinguishing malignant from benign conditions [101], while emerging markers such as circulating tumor cells, microRNAs, and exosomes show promise in refining risk assessment and guiding personalized treatment strategies [102].

The integration of biomarker-driven strategies in SN-38 and immunotherapy combinations offers a promising avenue for precision oncology. Biomarkers such as PD-L1, FOXO3, and tumor immune signatures could aid in identifying patients most likely to benefit from combination therapies, particularly those involving SN-38 and ICIs [103]. Stratifying patients based on molecular profiles has already transformed oncology, exemplified by the classification of breast cancer subtypes based on HER2, estrogen receptor (ER), and progesterone receptor (PR) expression. This approach is particularly crucial for TNBC, an aggressive subtype characterized by the absence of ER, PR, and HER2, where biomarker-guided strategies are essential for improving therapeutic outcomes [104]. Biomarker-driven treatment selection also plays a pivotal role in accelerating drug development by streamlining clinical trials and reducing costs [105]. The ability to stratify patients based on prognostic and therapeutic biomarkers has revolutionized cancer treatment, allowing for more individualized and effective interventions. As research advances, continued efforts in biomarker discovery and validation will be essential for fully integrating these strategies into SN-38-based therapies and immunotherapy regimens, paving the way for more precise, effective, and patient-specific cancer treatments.

### 5.4. Toxicity, Side Effects and Resistance to Combination Therapy

Despite the promising therapeutic potential of SN-38 and immunotherapy combinations in breast and prostate cancer treatment, toxicity and resistance remain major challenges. SN-38 is associated with dose-limiting toxicities, including gastrointestinal disturbances and myelosuppression, which can significantly impact patient tolerability [106]. When combined with ICIs, the risk of immune-related adverse events (irAEs) further complicates treatment, as ICIs can trigger multi-organ toxicities, affecting endocrine, gastrointestinal, and cardiovascular systems [107,108]. In particular, immune-mediated cardiotoxicity, although rare, can be severe and may present within weeks of ICI initiation [109]. The potential for additive or synergistic toxicities between SN-38 and ICIs underscores the need for careful patient monitoring and optimized dosing strategies.

A key consideration in managing toxicity is the temporal discrepancy between chemotherapy- and immunotherapy-induced adverse effects. While SN-38-related toxicities often emerge early in treatment, ICI-associated toxicities can develop unpredictably, sometimes occurring months after initiation [109]. This necessitates long-term monitoring and tailored supportive care strategies. Current research is focused on identifying predictive biomarkers, such as cytokines, human leukocyte antigens, and circulating antibodies, to stratify patients based on their risk of developing severe toxicities [108]. Such biomarker-driven approaches could enable personalized treatment regimens that maximize therapeutic efficacy while minimizing adverse effects.

In addition to toxicity concerns, resistance to SN-38 and immunotherapy combinations remains a significant barrier to clinical success. Breast and prostate cancers exhibit substantial heterogeneity, contributing to the development of adaptive resistance mechanisms [33,110]. In TNBC, resistance often arises from immune evasion strategies, including downregulation of tumor-specific antigens, deficiencies in antigen presentation, and failure to initiate an effective immune response [110]. These factors, combined with the activation of immunosuppressive signaling pathways, create a hostile TME that limits the effectiveness of combination therapies [110,111].

Similarly, prostate cancer presents unique challenges due to its low tumor mutational burden and inherently immunosuppressive TME, which contribute to poor immunotherapy responsiveness [33]. Overcoming resistance in this setting requires a deeper understanding of immune escape mechanisms and the development of novel combination strategies. Emerging research suggests that combining SN-38 with epigenetic modulators, metabolic inhibitors, or next-generation ICIs may help restore immune sensitivity and improve treatment efficacy. To address these challenges, future studies should focus on integrating biomarker-driven patient stratification, optimizing drug delivery through nanoformulations, and developing adjunctive agents capable of disrupting resistance pathways [33,110]. A comprehensive approach that incorporates precision medicine, immunomodulation, and innovative drug formulations holds the potential to enhance the therapeutic index of SN-38 and immunotherapy, ultimately improving patient outcomes in breast and prostate cancers.

### 5.5. Expanding the Applications of SN-38 and Immunotherapy Combinations

The integration of SN-38 with immunotherapy is rapidly evolving, showing promise not only in breast and prostate cancers but also in other malignancies such as ovarian and hepatocellular cancers. This expanding therapeutic landscape is driven by the potential of SN-38 as both a potent cytotoxic agent and an immunomodulatory enhancer. In breast cancer, the combination of SN-38-based ADCs with ICIs is being explored to enhance immune activation and therapeutic efficacy [112]. Given the traditionally immunosuppressive TME of breast tumors, these combinations could help transform them into more immunogenic targets, thereby improving patient responses to immunotherapy. In prostate cancer, where immunotherapy has historically shown limited success, combining SN-38 delivery systems with ICIs presents a novel avenue for investigation. This strategy aims to counteract the immunosuppressive TME that has hindered the effectiveness of immunotherapy in prostate cancer [35]. Additionally, SN-38-based therapies are being evaluated in combination with emerging immunotherapeutic modalities, such as bispecific T cell-engaging antibodies and chimeric antigen receptor (CAR)-T cell therapies, to enhance anti-tumor immune responses in both breast and prostate cancers [32,59].

Beyond these malignancies, SN-38’s versatility is gaining attention in other cancer types. Investigations into its integration with novel immunotherapeutics, including bispecific antibodies and CAR-T cells, could expand its application across a broader spectrum of tumors. The overarching goal is to leverage the distinct mechanisms of action of SN-38 and various immunotherapeutic agents to achieve more durable responses and overcome resistance mechanisms. As ongoing clinical trials continue to explore these combinations, their potential application to earlier disease stages and additional cancer types remain an area of active research. Continued innovation in SN-38-based immunotherapy strategies could unlock new frontiers in cancer treatment, broadening the scope of its clinical impact across multiple malignancies.

## 6. Future Perspective and Conclusions

While SN-38 is a potent chemotherapeutic agent with well-documented cytotoxic activity against proliferating tumor cells, its potential effects on immune cells, particularly activated lymphocytes, remain largely unexplored. Current literature provides limited direct evidence regarding clinically relevant concentrations of SN-38 spare or impair immune cell viability and function. Given the increasing interest in combining SN-38 with immune checkpoint inhibitors and other immunotherapies, this represents an important knowledge gap. Future studies should specifically investigate the impact of SN-38 on T-cell survival, activation, and effector functions, as well as its influence on antigen-presenting cells within the tumor microenvironment. Such work will be essential to optimize dosing regimens that preserve antitumor immunity while maximizing cytotoxic efficacy. The combination of SN-38, a potent topoisomerase I inhibitor, with immunotherapy represents a promising therapeutic strategy to overcome resistance and enhance antitumor efficacy. While SN-38 effectively induces DNA damage and apoptosis, its immunomodulatory effects, including the release of tumor-associated antigens, can synergize with immune checkpoint inhibitors to restore and amplify T-cell–mediated cytotoxicity. This dual approach will not only augment tumor clearance but also has the potential to induce durable and long-lasting immune responses in the patients. However, further studies are required to elucidate the molecular mechanisms underlying this synergy, optimize the dosing strategies, and evaluate the safety profiles in preclinical and clinical settings. Overall, the integration of SN-38 with immunotherapy could pave the way for more effective and personalized cancer treatment strategies.

## Data Availability

No new data were created or analyzed in this review. Data sharing is not applicable to this article.

## Abbreviations

The following abbreviations are used in this manuscript:

PCa	prostate cancer
ER	estrogen receptor
AR	androgen receptor
TME	tumor microenvironment
UGT1A1	UDP-glucuronosyltransferase 1A1
Nal-IRI	Nanoliposomal irinotecan
ADCs	antibody-drug conjugates
TNBC	triple-negative breast cancer
TILs	tumor-infiltrating lymphocytes
HR+	hormone receptor-positive
CRPC	castration-resistant prostate cancer
mTNBC	metastatic triple-negative breast cancer
ICI	immune checkpoint inhibitor
BiTEs	bispecific T-cell engagers
DAMPs	damage-associated molecular patterns
ICD	immunogenic cell death
HMGB1	high mobility group box 1
CTL	cytotoxic T lymphocyte
CRT	Calreticulin
APCs	antigen-presenting cells
NK	Natural killer
HNSCC	head and neck squamous cell carcinoma
Tregs	regulatory T cells
LPS	Lipopolysaccharide
APM	antigen-processing machinery
ICIs	immune checkpoint inhibitors
TROP-2	trophoblast cell surface antigen 2
ROS	reactive oxygen species
aPDL1	anti-PD-L1
CED	convection-enhanced delivery
PDX	patient-derived xenograft
HAS	human serum albumin
HA	hyaluronic acid
PSA	Prostate-specific antigen
PR	progesterone receptor
IrAEs	immune-related adverse events
